# Contrasting Aquaculture Systems Shape Distinct Growth and Short-Term Stress-Resistance Trait Clusters in the Red Swamp Crayfish

**DOI:** 10.3390/ani16081217

**Published:** 2026-04-16

**Authors:** Gao Gao, Lingyu Gan, Jingnan Wei, Hong Luo, Huiying Wang, Jialong Chen, Xiaoyi Su, Zhangxiu Li, Baoliang Bi, Dan Jia

**Affiliations:** 1 College of Animal Science and Technology, Yunnan Agricultural University, Kunming 650500, China; gaogao@ynau.edu.cn (G.G.); 18213608964@163.com (L.G.); w114425285@163.com (J.W.); 2023210428@stu.ynau.edu.cn (H.W.); 2024210436@stu.ynau.edu.cn (J.C.); 15287548771@163.com (X.S.); 18487176986@163.com (Z.L.); jiadan@ynau.edu.cn (D.J.); 2 Key Laboratory of Plateau Fishery Resources Protection and Sustainable Utilization, Universities of Yunnan Province, Kunming 650500, China; 3 Xishuangbanna Xinheng Ecological Agriculture Technology Co., Ltd., Xishuangbanna 666100, China; 13600068687@139.com; 4 International College, Yunnan Agricultural University, Kunming 650500, China

**Keywords:** *Procambarus clarkii*, rice–crayfish co-culture, phenotypic plasticity, trade-offs, intensive aquaculture

## Abstract

This study investigated whether these contrasting environments are associated with the development of coherent, organism-wide phenotypic–physiological syndromes reflecting a fundamental life-history trade-off. A controlled 60-day trial was conducted comparing crayfish from a high-input pond system (fed to satiation) and a low-input rice paddy system (primarily natural diet). Pond crayfish were fed a commercial formulated feed twice daily to satiation (approximately 3% of biomass per day). Rice paddy crayfish primarily consumed natural food webs and received a once-daily supplement of 30% of the pond feed amount (approximately 0.9% of biomass per day). Results revealed two distinct syndromes. Pond-cultured crayfish exhibited a growth-oriented syndrome: they were 33% heavier with a compact, volumetrically enhanced body shape, coupled with elevated lipid-anabolic enzyme activities (lipase and ACC), higher hemolymph protein and cholesterol concentrations, but also significantly increased levels of a stress-related endocrine factor (cortisol-like immunoreactivity) and oxidative damage (MDA). Conversely, rice paddy-cultured crayfish displayed a stress-resistance-oriented syndrome: a streamlined, deeper-abdomen morphology, fortified antioxidant (SOD, CAT, and GST) and immune (lysozyme and phenoloxidase) capacities, enhanced carbohydrate digestion (amylase), and lower systemic stress. Transcriptomic analysis showed that the hepatopancreas of paddy-cultured crayfish was enriched in pathways related to lipid metabolism, detoxification, and endocrine regulation, notably with upregulation of the *SULT1E1* gene. Our findings demonstrate that the distinct environmental and nutritional conditions of each aquaculture system are linked to specific multi-level adaptation syndromes, presenting a clear trade-off between rapid biomass production and systemic stress resistance (within the 60-day trial period), providing a mechanistic basis for optimizing sustainable practices: integrated systems enhance stock robustness, while intensive systems require strategies to mitigate physiological load.

## Simple Summary

The red swamp crayfish is a valuable food source often farmed in either intensively managed ponds or integrated rice field systems. This study investigated how these two very different farming environments affect the overall health and biology of the crayfish. We found that each system promotes a distinct set of coordinated traits, or a “syndrome.” Crayfish from ponds grew larger and heavier, prioritizing rapid biomass production. However, this came at a cost, as they also showed signs of higher physiological stress. In contrast, crayfish from rice paddies were smaller but possessed a stronger natural defense system, with enhanced antioxidant and immune capacities, suggesting they were more resilient to environmental challenges. This reveals a fundamental trade-off between fast growth and robust health depending on the farming method. Our findings are valuable for developing more sustainable aquaculture practices by highlighting that while intensive ponds maximize production, integrated rice-field systems produce hardier animals, and each system requires different management strategies to optimize both yield and animal welfare.

## 1. Introduction

The red swamp crayfish (*Procambarus clarkii*) is a globally significant freshwater crustacean, supporting a vast aquaculture industry due to its fast growth rate, high adaptability, and economic value [1]. Native to northeastern Mexico and the south–central United States, this species has been introduced to all continents except Antarctica and is now considered one of the most successful invasive aquatic organisms worldwide, largely owing to its high fecundity, broad environmental tolerance, and flexible feeding habits. In pursuit of higher productivity, traditional intensive pond monoculture has been widely adopted, characterized by high stocking densities, controlled environments, and formulated feed inputs aimed at maximizing biomass yield [2].

Concurrently, driven by principles of sustainability and ecological integration, rice–crayfish co-culture systems have gained substantial traction, particularly in Asia [3]. In such systems, crayfish utilize natural food webs within rice paddies (e.g., plankton, detritus, and insects), reducing reliance on external feed while offering ecosystem services such as pest control and nutrient recycling [4,5]. Recent studies have further highlighted the environmental and economic benefits of rice–crayfish integration, including improved water quality, reduced pesticide use, and enhanced land-use efficiency [6,7,8]. Specifically for *P. clarkii*, integrated rice–crayfish systems have been shown to influence growth performance, nutritional quality, and physiological resilience [4,5,9]. Moreover, integrated approaches have been successfully applied in other aquaculture sectors, such as cage fish farming in the Caspian Sea [10], land-based integrated multi-trophic aquaculture (IMTA) [11], and biofloc technology [12], providing valuable models for sustainable intensification. The choice between these divergent production systems—one prioritizing output and the other system resilience—represents a fundamental strategic decision in modern aquaculture, with direct implications for resource use, environmental impact, and animal welfare [13].

Existing research has begun to delineate some gross differences between intensive and integrated culture systems. Studies typically report that pond-cultured crustaceans achieve superior individual growth rates and final harvest weights, a direct benefit of optimized, nutrient-dense feeding regimes [9,13]. In the Pacific white shrimp (*Litopenaeus vannamei*), selective breeding for faster growth has been shown to simultaneously reduce stress tolerance, directly demonstrating a growth–resilience trade-off [4]. Similarly, in the Chinese mitten crab (*Eriocheir sinensis*), a reciprocal transplant experiment between pond and rice-field systems revealed that crabs from the rice-field environment exhibited higher antioxidant enzyme activities but lower growth rates, providing empirical support for environment-specific trade-offs [9]. In *Procambarus clarkii*, a feeding trial with varying dietary protein levels demonstrated that high-protein diets promoted growth but increased oxidative stress markers, further corroborating the growth–stress trade-off observed in our study [14]. In contrast, organisms from polyculture or integrated systems often exhibit different growth patterns and may demonstrate enhanced survival under disease challenge, suggesting a trade-off between maximum growth and robustness [15,16]. For instance, research on other aquatic species in integrated systems has highlighted improved water quality parameters and reduced disease incidence [17,18]. Furthermore, environmental complexity, such as that found in vegetated rice fields, has been shown to influence behavior and reduce aggression in cultured decapods, potentially lowering stress [19]. However, the current evidence remains largely compartmentalized, focusing on isolated metrics of production, environmental parameters, or specific stress responses without a unifying framework.

A critical knowledge gap persists in understanding the holistic biological adaptation of crayfish to these fundamentally different rearing environments. Specifically, it remains unclear whether the distinct selection pressures of a high-input pond versus a complex, low-input rice paddy ecosystem drive the development of coherent, organism-wide integrated phenotypes that encompass both morphological and physiological characteristics. Life-history theory predicts that organisms face trade-offs in allocating limited resources between traits promoting growth, reproduction, maintenance, and survival [20]. An environment favoring rapid biomass gain (like intensive ponds) may select for a “growth-oriented” phenotype, potentially at the expense of investment in stress resilience mechanisms [21]. Conversely, a more variable, resource-limited environment (like rice paddies) may favor a “resilience-oriented” phenotype with enhanced capabilities for environmental coping [22]. Yet, this theoretical framework has rarely been tested in commercially cultured crustaceans through an integrated analysis spanning morphology, systemic physiology, and underlying molecular regulation.

Therefore, this study was designed to investigate the multi-level adaptive responses of *P. clarkii* reared in traditional intensive pond monoculture and integrated rice–crayfish co-culture. It is important to acknowledge that the two aquaculture systems compared herein—intensive pond monoculture and integrated rice–crayfish co-culture—represent distinct combinations of production practices. They differ not only in environmental complexity (e.g., presence of rice plants, water flow) but also, and fundamentally, in nutritional input levels and sources (high-input formulated feed vs. a low-input natural diet). Consequently, the observed phenotypic and physiological divergence cannot be solely attributed to system structure alone. Instead, it likely reflects the combined and interacting effects of the entire suite of environmental and nutritional conditions characteristic of each production model. This study, therefore, characterizes the integrated, organismal-level syndromes that emerge under these two commercially relevant production packages. Future research employing a fully crossed design with feeding-matched controls in both systems would be valuable to disentangle the specific contributions of nutrition and environmental structure to the observed trade-off. We hypothesized that the two systems would promote distinct phenotypic–physiological syndromes, reflecting a fundamental life-history trade-off. To test this, we conducted a controlled 60-day comparative trial. We then employed an integrated analytical approach, combining detailed morphometric analysis, assays of key hepatopancreatic digestive and antioxidant enzymes, profiling of hemolymph-based immune and stress indicators, and deep transcriptomic sequencing of the hepatopancreas, a central organ for metabolism, digestion, and detoxification [9]. By synthesizing data across these biological scales, our study aims to provide a mechanistic understanding of how the integrated conditions of different aquaculture systems are associated with the health, resilience, and overall adaptation of the red swamp crayfish, offering scientific insights for the future development of optimized and sustainable production strategies. We acknowledge that a 60-day trial captures early physiological divergence but does not assess long-term adaptation or resilience.

## 2. Materials and Methods

### 2.1. Animals and Sample Collection

**Figure 1:**
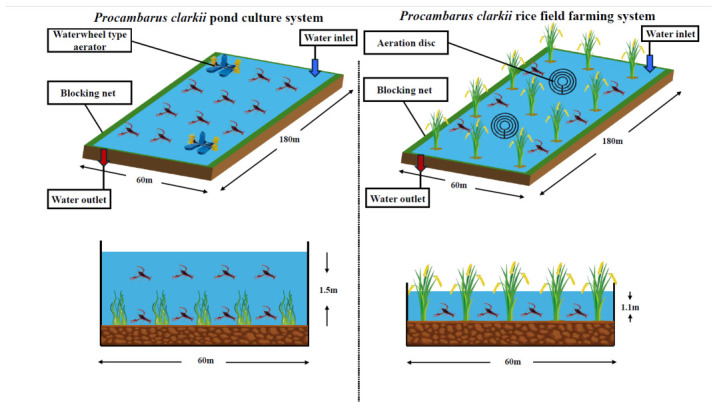
Schematic diagram of the two aquaculture systems used in this study. (**Left**) Traditional intensive pond monoculture system, featuring aeration discs and a waterwheel aerator. (**Right**) Integrated rice–crayfish co-culture system, with blocking nets and natural vegetation. Both systems were established in Mengzhe Town, Xishuangbanna, Yunnan Province, China.

This study conducted a 60-day comparative growth trial in Mengzhe Town, Xishuangbanna, Yunnan Province, China, to evaluate the effects of different aquaculture systems on crayfish. A 60-day period was selected because it covers the critical early post-juvenile phase when growth and physiological differentiation are most responsive to environmental conditions, and it is consistent with previous comparative studies on crustacean aquaculture systems. Uniform juvenile crayfish (initial body weight 5.0 ± 0.5 g) were obtained from a local commercial hatchery. After a one-week acclimation period, they were randomly assigned to two experimental groups: a traditional intensive pond monoculture system (Pond group, C) and a rice–crayfish integrated system (Rice field group, T), as illustrated in Figure 1. Each group consisted of three independent replicate units (six replicates in total), and all replicates were stocked at a standardized density of 15 individuals per square meter. During the trial, group C was fed a commercial formulated feed twice daily (08:00 and 17:00) to satiation, with a feeding rate of approximately 3% of the estimated crayfish biomass. Group T primarily relied on the natural productivity of the rice field ecosystem (plankton, detritus, and benthos) and was supplemented once daily (17:00) with 30% of the same commercial feed amount as group C (equivalent to approximately 0.9% of the biomass), simulating a common low-input co-culture model. The commercial formulated feed used for the pond group (and as a 30% daily supplement for the rice paddy group) was produced by Tongwei Co., Ltd. (Harbin, China) under the product name ‘Crayfish Special Compound Feed No. 2’. The feed was presented as 2.0 mm diameter sinking pellets. Its proximate composition (as determined by the manufacturer and verified by our laboratory using AOAC methods) was as follows (on a dry matter basis): crude protein 32.5 ± 0.6%, crude lipid 6.8 ± 0.3%, crude fiber 5.2 ± 0.4%, ash 12.0 ± 0.5%, moisture 10.0 ± 0.2%, and nitrogen-free extract (calculated by difference, representing mainly digestible carbohydrates) 33.5 ± 1.0%. The main ingredient list included fish meal, soybean meal, rapeseed meal, wheat flour, rice bran, fish oil, soybean lecithin, vitamin and mineral premix (vitamin A, D3, E, K3, B1, B2, B6, B12, niacin, pantothenic acid, folic acid, biotin, choline chloride, FeSO_4_, CuSO_4_, MnSO_4_, ZnSO_4_, CoCl_2_, Na_2_SeO_3_, and Ca(IO_3_)_2_), and an antioxidant (ethoxyquin). The natural food web available to crayfish in the rice paddy system was characterized through weekly sampling (three random sites per replicate) during the 60-day trial. Major components included the following: (i) plankton (dominated by chlorophytes such as *Chlorelal* spp. and *Scenedesmus* spp. and diatoms such as *Navicula* spp.), (ii) detritus derived from decomposed rice straw and leaf litter, (iii) benthic macroinvertebrates (mainly chironomid larvae, oligochaetes, and gastropods), and (iv) periphyton attached to rice stems. Based on literature values for similar rice–crayfish systems [4] and our own proximate analysis of pooled natural diet samples (*n* = 9, three per replicate), the estimated nutritional profile of the natural diet was as follows: crude protein 16.3 ± 2.1%, crude lipid 3.7 ± 0.8%, crude fiber 14.5 ± 1.8%, ash 18.2 ± 2.0%, and carbohydrates (by difference) 47.3 ± 3.2%. Detailed composition of each natural food component is provided in Supplementary Table S4. Water quality parameters (water temperature 26.5 ± 2.5 °C, dissolved oxygen >5.0 ± 0.8 mg/L, pH 7.6 ± 0.4, ammonia nitrogen <0.3 ± 0.05 mg/L) were monitored weekly to ensure both systems remained within the optimal range for crayfish culture.

Crayfish were harvested at the end of the 60-day culture period. For morphological analysis, 30 individuals were randomly sampled from each replicate (*n* = 90 per group), anesthetized on ice, and measured for body length, total length, body weight, and detailed carapace and abdomen dimensions. Following morphological measurements, tissue and hemolymph samples were collected from a subset of specimens. For transcriptomic and enzymology analyses, hepatopancreas tissues from 6 individuals per replicate (*n* = 18 per group) were rapidly dissected, flash-frozen in liquid nitrogen, and stored at −80 °C for subsequent RNA extraction and biochemical assays. For hemolymph analysis, an additional 10 individuals from each of the C and T groups (*n* = 10 per group) were randomly selected. Hemolymph was collected using a pre-chilled sterile 1 mL syringe containing an anticoagulant (10 mM EDTA, 0.1 M sodium citrate, pH 7.0), immediately placed on ice, and centrifuged (4 °C, 3000× *g*, 10 min) to collect the supernatant for subsequent immune and stress parameter analysis.

### 2.2. Morphological Measurement and Data Analysis

Following sample collection, the key morphometric parameters (body length, total length, body weight, carapace length/width/height, and abdominal length/width/height) were measured using digital calipers (Mitutoyo Corporation, Kawasaki, Japan; precision 0.01 mm) and an electronic balance (Mettler Toledo, Columbus, OH, USA; precision 0.01 g). Derived shape indices, including Carapace Width/Length Index, Carapace Height/Length Index, Abdominal Width/Length Index, and Abdominal Height/Length Index, were calculated by dividing the corresponding width or height by the length. All data are presented as mean ± standard error (SE).

### 2.3. RNA Extraction, Library Construction, and Transcriptome Sequencing

Total RNA was extracted from the hepatopancreas samples (*n* = 6 per replicate, 18 per group) using TRIzol reagent (Invitrogen, Carlsbad, CA, USA) following the manufacturer’s protocol. RNA integrity was assessed using an Agilent 2100 Bioanalyzer (Agilent Technologies, Santa Clara, CA, USA), and samples with an RNA Integrity Number (RIN) ≥ 7.0 were used for subsequent analysis. Sequencing libraries were constructed using the NEBNext^®^ Ultra™ RNA Library Prep Kit for Illumina^®^ (New England Biolabs, Ipswich, MA, USA) according to the manufacturer’s instructions. Briefly, mRNA was purified from total RNA using poly-T oligo-attached magnetic beads, fragmented, and reverse transcribed into first-strand cDNA. Second-strand cDNA synthesis was followed by end repair, adenylation of 3′ ends, adapter ligation, and PCR amplification. The quality of the libraries was checked using an Agilent Bioanalyzer 2100 system. The libraries were then sequenced on an Illumina NovaSeq 6000 platform by Annoroad Gene Technology (Beijing) Co., Ltd. (Beijing, China) to generate 150 bp paired-end reads.

### 2.4. Transcriptomic Data Processing and Differential Expression Analysis

Raw sequencing reads were processed to obtain clean data by removing reads containing adapters, reads with poly-N sequences, and low-quality reads using fastp (version 0.20.0). The clean reads were then mapped to the *Procambarus clarkii* reference genome (assembly ASM308452v1) using HISAT2 (version 2.1.0). FeatureCounts (version 1.6.3) was used to count the read numbers mapped to each gene. Principal component analysis (PCA) was performed using the ‘stats’ package (built-in, R version 4.0.5) to visualize sample clustering. Differential expression analysis between the two groups was conducted using the DESeq2 R package (version 1.26.0). Genes with an adjusted *p*-value (padj) < 0.05 and an absolute log2 fold change ≥ 0.585 (equivalent to a 1.5-fold change) were considered differentially expressed genes (DEGs). A Venn diagram was generated to visualize shared and unique genes between groups.

### 2.5. Functional Enrichment Analysis

Gene Ontology (GO) enrichment analysis and Kyoto Encyclopedia of Genes and Genomes (KEGG) pathway enrichment analysis of the DEGs were performed using the clusterProfiler R package (version 3.14.3). GO terms and KEGG pathways with a corrected *p*-value (*q*-value) < 0.05 were considered significantly enriched. The analysis results were visualized using ggplot2.

### 2.6. Hepatopancreatic Enzyme Activity Assays

Hepatopancreas samples were homogenized in ice-cold phosphate-buffered saline (PBS, 0.1 M, pH 7.4). The homogenates were centrifuged at 10,000× *g* for 15 min at 4 °C, and the resulting supernatants were collected for enzymatic assays. The activities of superoxide dismutase (SOD), catalase (CAT), glutathione S-transferase (GST), total lipase, amylase, and acetyl-CoA carboxylase (ACC) were measured using commercially available assay kits (Nanjing Jiancheng Bioengineering Institute, China) following the manufacturer’s protocols. All enzymatic activities were normalized to the total protein content of the supernatant, which was determined using a Bradford protein assay kit (Beyotime Biotechnology, Shanghai, China).

### 2.7. Hemolymph Parameter Analysis

The collected hemolymph supernatant was used for the analysis of physiological and immune parameters. Total protein, glucose, total cholesterol, and malondialdehyde (MDA) concentrations were determined using commercial colorimetric assay kits (Nanjing Jiancheng Bioengineering Institute, Nanjing, China). The activities of lysozyme and phenoloxidase (PO) were measured using kinetic assay kits (Shanghai Enzyme-linked Biotechnology Co., Ltd., Shanghai, China) based on the lysis of Micrococcus lysodeikticus and the oxidation of L-dihydroxyphenylalanine (L-DOPA), respectively. Cortisol concentration was quantified using a commercially available enzyme-linked immunosorbent assay (ELISA) kit (Cusabio Technology LLC, Wuhan, China) specific for crustaceans. All assays were performed according to the manufacturers’ instructions.

### 2.8. Integrative Statistical Analysis

To synthesize the multi-level data, an integrative analysis of key morphological and physiological traits was conducted. A Spearman correlation matrix was calculated among all measured parameters (e.g., body weight, shape indices, enzyme activities, and hemolymph parameters) using the R package ‘corrplot’ to visualize the correlation patterns. Hierarchical clustering of individual crayfish samples based on the standardized values (z-scores) of these integrated traits was performed using the ‘pheatmap’ R package, with Euclidean distance as the dissimilarity metric and the complete linkage method. The clustering aimed to visualize the distinct phenotypic–physiological syndromes associated with each culture environment.

To synthesize the multi-level data and delineate the overarching trait associations, an integrative correlation and clustering analysis was performed on key quantifiable phenotypic and physiological traits across all individual crayfish. A Spearman rank correlation matrix was calculated among the measured parameters, which included morphological traits (Body weight, Carapace Width/Length index, Carapace Height/Length index, Abdominal Width/Length index, Abdominal Height/Length index), hepatopancreatic enzyme activities (Superoxide dismutase-SOD, Catalase-CAT, Glutathione S-transferase-GST, Total lipase, Amylase, Acetyl-CoA carboxylase-ACC), and hemolymph parameters (Total protein, Total cholesterol, Lysozyme activity, Cortisol, Malondialdehyde-MDA). The correlation analysis was conducted using the R package ‘corrplot’ to visualize the strength and direction (positive or negative) of the pairwise relationships, as detailed in the correlation matrix. Subsequently, hierarchical clustering of individual crayfish samples was performed based on the standardized values (z-scores) of this integrated trait set using the ‘pheatmap’ R package. Euclidean distance was used as the dissimilarity metric with the complete linkage method. This dual approach aimed to statistically validate and visually represent the distinct phenotypic–physiological syndromes associated with each culture environment. The correlation matrix revealed a structured pattern of trait covariation, while the clustering analysis demonstrated the segregation of individuals based on their culture system.

### 2.9. Statistical Analysis

All statistical analyses were performed using SPSS software (version 26.0, IBM Corp, Armonk, NY, USA) and R (version 4.0.5) with appropriate packages. For comparisons between the pond (C) and rice-paddy (T) groups, independent samples *t*-tests were used for morphological parameters, hepatopancreatic enzyme activities, and hemolymph parameters. A significance threshold of *p* < 0.01 was applied unless otherwise indicated. Multivariate analyses (Spearman correlation and hierarchical clustering) were performed as described in Section 2.8.

## 3. Results

### 3.1. Morphological Divergence Between Pond and Paddy-Cultured Crayfish

Morphometric analysis revealed distinct phenotypic trajectories in *Procambarus clarkii* shaped by the pond (C group) and rice–crayfish co-culture (T group) environments in Xishuangbanna. Crayfish in the C group demonstrated a growth strategy oriented towards rapid biomass accumulation. They attained significantly greater body length, total length, and body weight compared to the T group (*p* < 0.001), with body weight being 33.0% higher (Table 1). This was accompanied by a compact and volumetrically enhanced body plan: a shorter but wider and taller carapace (higher Carapace Width/Length and Height/Length indices, *p* < 0.001, Table 2) and a wider, flatter abdomen (higher Abdominal Width/Length Index but lower Height/Length Index, *p* < 0.001, Table 3). In contrast, crayfish from the T group exhibited a morphology indicative of adaptation to a complex agro-ecosystem. Despite lower absolute mass, they developed a significantly longer and more streamlined carapace (lower Carapace Width/Length and Height/Length indices, Table 2), a trait that may enhance maneuverability within the structured rice paddy habitat. Concurrently, they possessed a deeper and more robust abdomen (higher Abdominal Height/Length Index, Table 3), which could be associated with stronger swimming musculature and potentially greater investment in physiological reserves for coping with environmental variability. Thus, the phenotypic divergence reflects a fundamental trade-off: the C group’s morphology is optimized for efficient growth in high-input, controlled ponds, whereas the T group’s morphology suggests an investment in traits favoring ecological performance, resilience, and fitness within the integrated and variable rice field environment.

### 3.2. Transcriptomic Analysis Reveals Hepatopancreatic Adaptation in Crayfish

**Figure 2:**
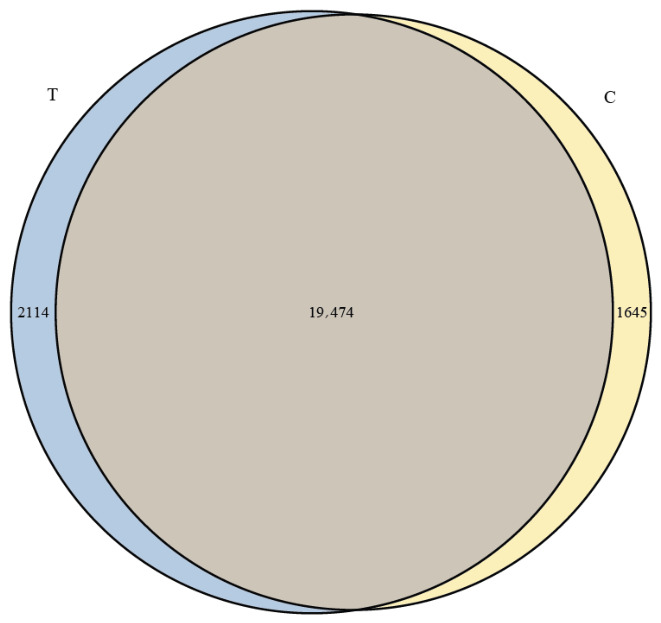
Venn diagram of expressed genes in the hepatopancreas of crayfish from RFC (T) and pond-cultured (C) groups.

**Figure 3:**
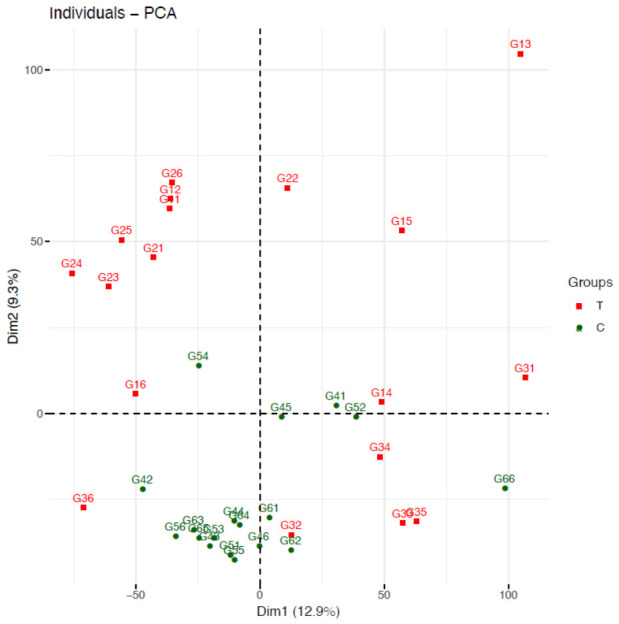
Principal component analysis (PCA) of hepatopancreas transcriptomes from the T and C groups.

**Figure 4:**
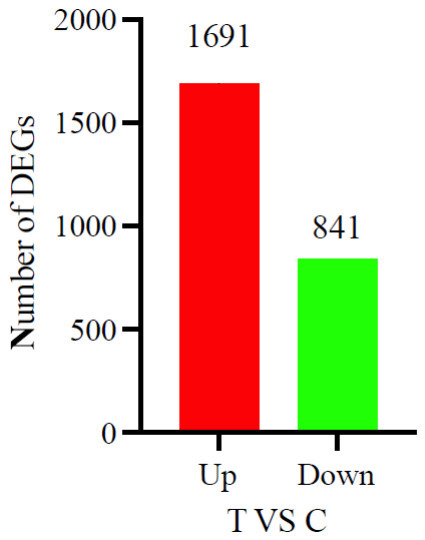
Statistics of differentially expressed genes (DEGs) between the T and C groups.

A total of 235 GB of clean data was obtained from both the C and T groups, with each sample reaching over 5.92 GB. The Q20 and Q30 base ratios exceeded 98.58% and 95.97%, respectively, and the GC content was above 42.77%. After filtering low-quality reads, a total of 1,526,574,210 clean reads were retained (detailed sequencing metrics are provided in Table S1). Venn diagram analysis showed that 23,233 genes were present across both groups, with 2114 genes unique to the T group and 1645 genes unique to the C group (Figure 2). Principal component analysis (PCA) of the transcriptomic data revealed clear separation between the two groups, indicating distinct transcriptional profiles influenced by the different rearing environments (Figure 3). Differential gene expression analysis (fold change ≥ 1.5, *p* < 0.05) identified 2532 differentially expressed genes (DEGs), of which 1691 were upregulated and 841 were downregulated (Figure 4).

**Figure 5:**
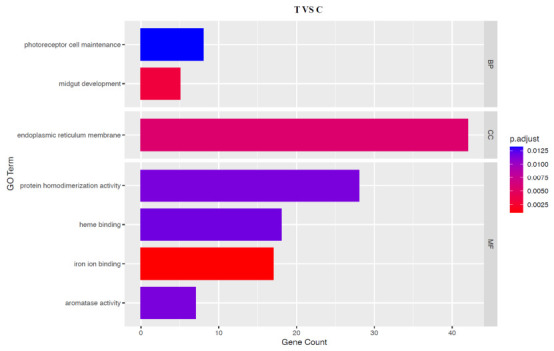
Gene Ontology (GO) enrichment analysis of the hepatopancreatic DEGs.

Our transcriptomic comparison revealed a substantial number of differentially expressed genes (DEGs) in the hepatopancreas of the T group (Table S2), providing the foundation for subsequent functional analysis. GO enrichment analysis of the hepatopancreatic DEGs delineated a coherent adaptive signature in response to the distinct culture environments (Figure 5). Significantly enriched terms converged on three interconnected biological themes. First, the enrichment of terms related to enhanced environmental sensing and adaptive tissue remodeling, such as ‘photoreceptor cell maintenance’ and ‘midgut development’, suggests that T-group crayfish have heightened perception of the variable light regime in rice paddies and are equipped with a digestive system potentially fine-tuned for the diverse natural diet available in this ecosystem. Second, enrichments were observed in categories fundamental to cellular architecture and functional complexes, such as ‘endoplasmic reticulum membrane’ and ‘protein homodimerization activity’. Finally, key metabolic and catalytic processes were highlighted, notably ‘heme binding’, ‘iron ion binding’, and ‘aromatase activity’. This specific functional profile suggests that the crayfish’s adaptation involves enhanced environmental perception, restructuring of digestive and cellular infrastructure, and reprogramming of core metabolic pathways, collectively providing a molecular substrate for systemic physiological adjustment.

**Figure 6:**
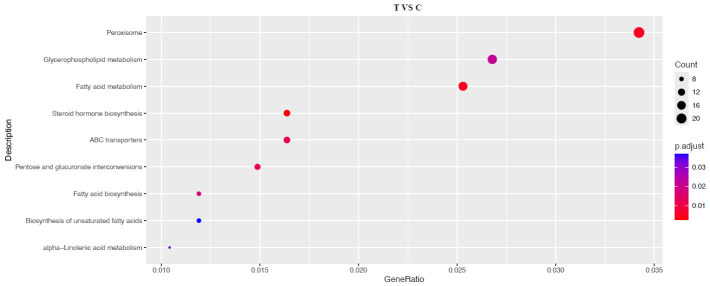
KEGG pathway enrichment analysis of the hepatopancreatic DEGs.

KEGG pathway enrichment analysis further substantiated the metabolic reprogramming in the hepatopancreas (Figure 6). The most significantly enriched pathways were predominantly associated with lipid and xenobiotic metabolism. Key categories included lipid metabolism and transport (‘Glycerophospholipid metabolism’, ‘Fatty acid metabolism’, ‘Fatty acid biosynthesis’, ‘Biosynthesis of unsaturated fatty acids’, ‘alpha-Linolenic acid metabolism’, and ‘ABC transporters’), specialized lipid-derived signaling (‘Steroid hormone biosynthesis’), and cellular metabolic organelles (‘Peroxisome’). The coordinated enrichment of these pathways delineates a comprehensive metabolic adaptation. The shift in lipid metabolism pathways may reflect an optimized utilization of plant-based and diverse lipid sources prevalent in rice fields. Concurrently, the activation of ‘Peroxisome’ and detoxification-related pathways (e.g., ‘ABC transporters’) underscores a fortified cellular capacity to maintain homeostasis under the more complex chemical milieu of an integrated agro-ecosystem.

**Figure 7:**
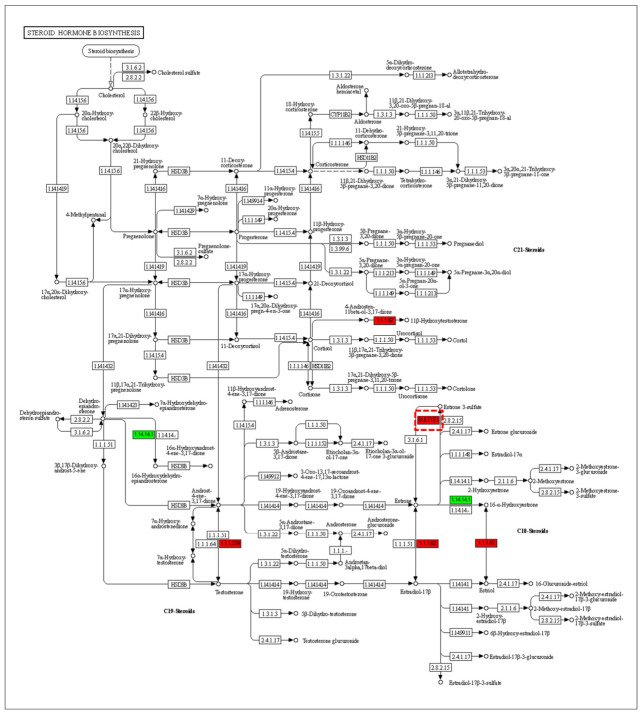
Summary of the steroid hormone biosynthesis pathway highlighting the key regulatory gene *SULT1E1*.

KEGG analysis further identified significant enrichment in the ‘Steroid hormone biosynthesis’ pathway, indicating a concurrent endocrine regulatory dimension to the adaptation (Table S3). Pathway mapping pinpointed a key differential node: the sulfotransferase family 1E member 1 (*SULT1E1*) gene, which was markedly upregulated in the T group (Figure 7). The marked upregulation of the *SULT1E1* gene points to a sophisticated endocrine adaptation. This enhancement in hormone sulfonation capacity likely enables more precise regulation of steroid hormone homeostasis, a crucial mechanism for modulating growth, development, and reproductive strategies in response to the distinct biotic and abiotic cues of the rice paddy environment. This regulatory fine-tuning represents a molecular cornerstone for the physiological resilience and adaptive fitness observed in the rice–crayfish co-culture system.

### 3.3. Enzymatic Activity Profile Reveals Differential Physiological Adaptations

The analysis of hepatopancreatic enzymatic activities further elucidated the physiological divergence between the two groups (Table 4). Crayfish from the T group exhibited significantly enhanced activities of key antioxidant enzymes, including superoxide dismutase (SOD), catalase (CAT), and glutathione S-transferase (GST) (*p* < 0.001 for all), indicating a fortified cellular defense system against oxidative stress. Concurrently, T-group crayfish displayed markedly higher amylase activity (*p* < 0.001), supporting an improved capacity to utilize carbohydrate-rich natural resources in rice paddies. In contrast, the C group showed significantly greater activities of lipase and acetyl-CoA carboxylase (ACC) (*p* < 0.001), enzymes central to lipid digestion and biosynthesis, aligning with their strategy of rapid biomass accumulation.

### 3.4. Hemolymphatic Profiles Reflect Differential Health and Stress Status

Analysis of hemolymph parameters revealed systemic physiological differences aligning with the distinct culture environments (Table 5). Crayfish from the T group exhibited a significantly more robust innate immune response, demonstrated by 56% and 51% higher activities of lysozyme and phenoloxidase, respectively (*p* < 0.001). Concurrently, they displayed 36% lower levels of malondialdehyde (MDA) (*p* < 0.001), indicating reduced oxidative damage, which corresponds with their enhanced hepatopancreatic antioxidant capacity. In contrast, the C group showed higher concentrations of total protein and total cholesterol (*p* < 0.001), consistent with their anabolic growth strategy, but also sustained elevated baseline levels of a stress-related endocrine factor (cortisol-like immunoreactivity) (*p* < 0.001), suggesting a higher cumulative physiological load under intensive pond conditions.

### 3.5. Integrative Correlation and Cluster Analyses Confirm Distinct Phenotypic–Physiological Syndromes

To synthesize the multi-level adaptations observed in morphology, hepatopancreatic function, and systemic physiology, we performed an integrative correlation and hierarchical cluster analysis on key quantifiable phenotypic and physiological traits across all individual crayfish. This analysis aimed to delineate the overarching trait associations that define the organismal-level response to each culture environment.

**Figure 8:**
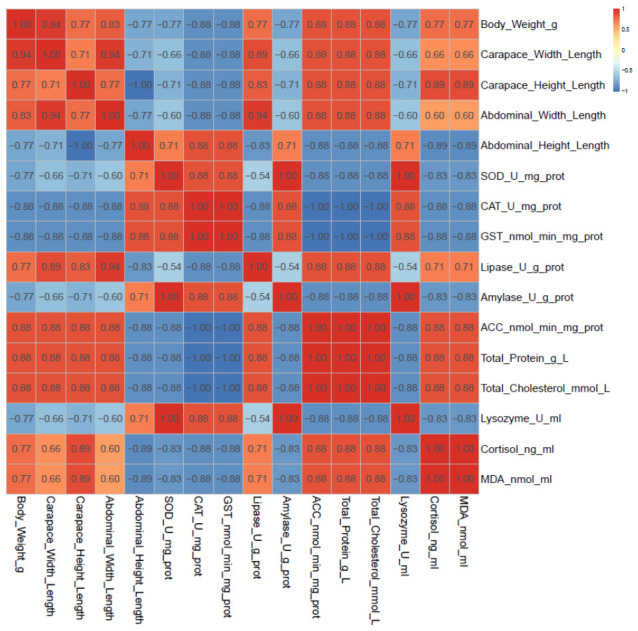
Heatmap of Spearman correlation coefficients among key phenotypic and physiological parameters. The color scale indicates the strength and direction of correlations, with red representing strong positive correlations and blue representing strong negative correlations.

The Spearman correlation matrix revealed a highly structured pattern (Figure 8). A strong, positive correlation network (red) unified the defining features of the pond-cultured (C group) phenotype: greater body weight, a compact body plan (high carapace and abdominal width-to-length indices), and physiological markers of a lipid-anabolic state (high lipase and ACC activity and elevated total protein and total cholesterol). This integrated module was strongly and negatively correlated (blue) with a suite of traits characteristic of the rice paddy-cultured (T group) phenotype. This opposing module showed strong internal positive correlations among a streamlined, robust morphology (high abdominal height-to-length index), enhanced antioxidant capacity (SOD, CAT, and GST activity), elevated carbohydrate digestion (amylase activity), and stronger innate immunity (lysozyme activity). Notably, markers of chronic stress (cortisol) and oxidative damage (MDA) clustered together and were positively correlated with the C group’s growth-lipid module but negatively correlated with the T group’s resilience module, statistically validating the physiological trade-off between rapid biomass production and homeostasis maintenance.

**Figure 9:**
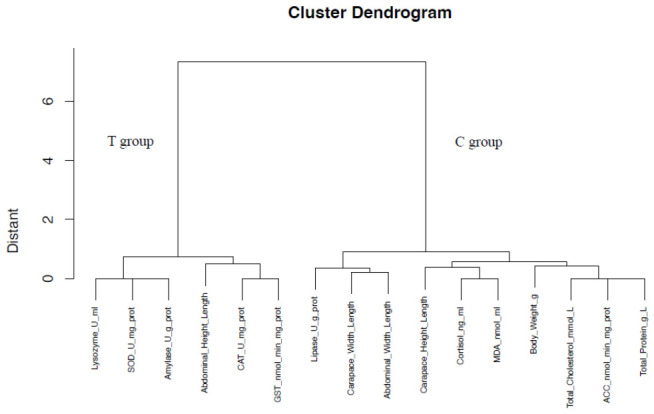
Hierarchical cluster dendrogram of crayfish samples based on integrated phenotypic–physiological traits. Samples are distinctly separated into two primary clusters corresponding perfectly to their culture system (Rice Paddy T1–T3 vs. Pond C1–C3). The *x*-axis represents sample names, and the *y*-axis represents the Euclidean distance. Samples with more similar trait profiles (higher correlation) cluster closer together.

Hierarchical clustering of samples based on this trait profile resulted in a perfect bifurcation, with all T-group and C-group individuals forming two distinct clades (Figure 9). Trait clustering further grouped the T-group-associated resilience traits (Lysozyme, SOD, Amylase, and Abdominal_Height_Length) into one major branch, while the C-group-associated growth and lipid metabolism traits (Body_Weight, Total_Cholesterol, ACC, and Total_Protein) clustered separately, with stress/damage markers (Cortisol and MDA) showing closer affinity to the latter.

Collectively, these multivariate analyses demonstrate that the two culture environments promote the development of two coherent, yet antagonistic, phenotypic–physiological syndromes. The pond environment selects for an integrated “Growth-Oriented” syndrome, characterized by a compact morphology, lipid-centric metabolism, and higher apparent physiological load. In contrast, the rice paddy environment promotes a stress-resistance-oriented syndrome, integrating a streamlined morphology, fortified antioxidant and immune defenses, enhanced carbohydrate utilization, and lower systemic stress. The organismal-level syndromes revealed by this analysis are consistent with and are mechanistically supported by the specific molecular adaptations observed in the hepatopancreas transcriptome, including the reprogramming of lipid and xenobiotic metabolism and the endocrine fine-tuning mediated by *SULT1E1* upregulation. Thus, the environmental adaptation in *P. clarkii* manifests as a coordinated restructuring across morphological, physiological, and molecular levels, presenting a fundamental trade-off between production efficiency and ecological fitness.

## 4. Discussion

This study elucidates the comprehensive and multi-level adaptive responses of *Procambarus clarkii* reared in two contrasting aquaculture environments. Our integrated results demonstrate that the conditions inherent to traditional intensive pond monoculture and integrated rice–crayfish co-culture are associated with the development of two distinct, coherent phenotypic–physiological syndromes. This divergence represents a fundamental biological trade-off driven by differential resource allocation: the pond environment, with abundant formulated feed, favors channeling energy towards rapid growth (lipid anabolism and protein synthesis) at the cost of elevated oxidative stress and chronic physiological load, whereas the rice paddy environment, with variable natural diets and structural complexity, selects for integrated traits that enhance nutrient utilization, detoxification, immune competence, and antioxidant defense. The syndromes are mechanistically underpinned by coordinated molecular reprogramming—exemplified by the upregulation of *SULT1E1* and pathways for peroxisome function and xenobiotic metabolism—which translates environmental cues into systemic physiological adjustments. Thus, the observed multi-trait integration reflects the functional interdependence of morphology, metabolism, and immunity under specific selection regimes, providing a holistic view of environmental adaptation in this commercially important species. The observed differences in digestive enzyme activities are directly interpretable in light of the contrasting dietary compositions. The pond-cultured crayfish were fed a formulated diet containing 6.8% crude lipid and 33.5% carbohydrates. The high lipid content (nearly double that of the natural diet) explains the significantly elevated lipase activity in the pond group (180.3 ± 5.1 vs. 124.2 ± 4.8 U/g protein, *p* < 0.001), as lipase is the primary enzyme responsible for triglyceride hydrolysis. Concurrently, the elevated acetyl-CoA carboxylase (ACC) activity (8.6 ± 0.4 vs. 5.0 ± 0.3 nmol/min/mg protein, *p* < 0.001) indicates enhanced de novo fatty acid synthesis from excess dietary carbohydrate and protein, a typical anabolic response to energy-rich formulated feeds. In contrast, the rice paddy-cultured crayfish consumed a natural diet with an estimated carbohydrate content of 47.3%, which is substantially higher than that of the formulated feed (33.5%). This carbohydrate-rich diet explains the markedly higher amylase activity in the paddy group (119.7 ± 3.5 vs. 84.8 ± 2.4 U/g protein, *p* < 0.001), as amylase is the key enzyme for starch and glycogen digestion. These diet–enzyme relationships are consistent with well-established physiological principles in decapod crustaceans [23,24] and strongly support the conclusion that nutritional input is a major driver of the metabolic divergence observed between the two systems.

The pronounced morphological divergence is the most direct evidence of environment-specific selection pressures. The compact, volumetrically enhanced body plan of pond-cultured crayfish, characterized by greater weight, a wider and taller carapace, and a flatter abdomen, is congruent with a strategy of maximizing energy storage and somatic growth under conditions of high, predictable nutrient input and reduced physical complexity. This morphology likely optimizes feed conversion efficiency, a primary goal in intensive aquaculture. In stark contrast, the morphology of paddy-cultured crayfish—featuring a streamlined carapace and a deeper, more robust abdomen—suggests an investment in traits crucial for survival in a spatially complex and variable habitat. This aligns with findings that different cultivation modes (pond vs. paddy) significantly influence morphological traits in *P. clarkii* [4]. The streamlined shape may enhance maneuverability for navigating dense rice stems, a form of habitat-driven morphological plasticity documented in other crayfish species, where body shape changes adapt to water flow and habitat structure [25]. Concurrently, the deeper abdomen indicates stronger swimming musculature, supporting greater locomotion for foraging and coping with environmental fluctuations such as water level changes. This morphological trade-off perfectly encapsulates the core compromise between maximizing growth efficiency in a controlled setting and investing in traits that enhance fitness and survival in a more challenging, heterogeneous ecosystem.

Underpinning these phenotypic differences is a profound metabolic and molecular reprogramming, primarily centered in the hepatopancreas. The transcriptomic and enzymatic data reveal that the adaptation is not superficial but involves a fundamental restructuring of core physiological processes. The growth-oriented syndrome is molecularly characterized by elevated activities of lipase and acetyl-CoA carboxylase (ACC), coupled with higher hemolymph cholesterol and protein concentrations. This confirms a metabolic state channeling resources towards lipid anabolism and protein synthesis for rapid growth. However, this high-turnover, anabolic state is linked to a significant physiological cost, as evidenced by elevated baseline levels of a stress-related endocrine factor (cortisol-like immunoreactivity) and malondialdehyde (MDA) in the hemolymph. Such a physiological state mirrors the energetic trade-offs observed in reproductive male crayfish, which prioritize energy allocation to growth and reproduction at the expense of higher oxidative stress [14]. Conversely, the short-term stress-resistance trait cluster-oriented syndrome exhibits a multifaceted molecular signature designed for homeostasis: enhanced antioxidant defenses (higher SOD, CAT, and GST), a metabolic shift towards carbohydrate utilization (higher amylase) likely reflecting adaptation to the detritus- and plant-based natural diet in paddies, and a fortified innate immune system (higher lysozyme and phenoloxidase). The upregulation of these antioxidant and immune pathways is a conserved response to environmental stress in *P. clarkii*, commonly observed under challenges like high temperature and pollutant exposure [26,27,28,29]. Critically, the transcriptome analysis uncovered a sophisticated endocrine adjustment, marked by the significant upregulation of the *SULT1E1* gene within the steroid hormone biosynthesis pathway [27]. This suggests an enhanced capacity for hormonal fine-tuning, potentially modulating energy allocation between growth, maintenance, and reproduction in response to the variable paddy environment. This regulatory plasticity is a key feature enabling invasive species like *P. clarkii* to thrive in diverse and sometimes polluted habitats, as population-specific transcriptional responses to stressors can indicate local adaptation [14]. Thus, the hepatopancreas serves as a central hub, integrating dietary, metabolic, and endocrine signals to orchestrate the systemic shift towards a short-term stress-resistance trait cluster-focused physiology [29].

The power of this study lies in the integration of data across biological scales. The multivariate correlation and cluster analyses statistically validate the existence of the two antagonistic trait modules and their perfect alignment with the rearing environment. The strong positive correlations within each syndrome—linking morphological traits, enzyme activities, and hemolymph parameters—demonstrate that these are cohesive, organismal-level strategies rather than isolated changes. The negative correlations between the modules, particularly the association of stress/damage markers with the growth module and their dissociation from the short-term stress-resistance trait cluster module, provide robust statistical evidence for the physiological trade-off between high productivity and homeostasis maintenance. This integrated view supports the concept that adaptive responses in crustaceans are systemic, involving coordinated changes from gene expression to whole-organism physiology [30,31]. The clear bifurcation of all individuals into environment-specific clusters in the hierarchical analysis underscores the potency of the cultivation system as a driver of phenotypic integration, a phenomenon observed in other aquatic species facing distinct ecological pressures [7].

The implications of these findings extend beyond basic biology into aquaculture practice and sustainability. For intensive pond systems, the results highlight a potential conflict between maximizing growth and maintaining animal health. The observed “Growth-Oriented Syndrome” with its markers of chronic stress suggests a need for management strategies (optimized stocking density, improved diet formulations, and environmental enrichment) that mitigate physiological load to ensure long-term sustainability, animal welfare, and potentially higher disease resistance. For integrated rice–crayfish systems, this research provides a physiological and molecular validation of their ecological value. The “short-term stress-resistance trait cluster -oriented syndrome” demonstrates that these systems produce animals with superior health indicators, robustness, and likely greater capacity to cope with disease and environmental fluctuation. This trade-off informs sustainable aquaculture development, where the choice of system may depend on whether the priority is maximum yield or product quality, system short-term stress-resistance trait cluster, and ecosystem services such as reduced pesticide use and improved water quality, which are documented benefits of rice–aquatic species integration [5,6,8]. Future research should investigate the long-term and transgenerational aspects of these syndromes, explore their direct consequences for disease resistance and survival under specific environmental challenges, and evaluate the potential of key molecular markers like *SULT1E1* or other stress-responsive genes identified here in selective breeding programs. Such programs could aim to develop genotypes that better balance growth and short-term stress-resistance trait cluster traits for different production environments, ultimately enhancing the sustainability and climate short-term stress-resistance trait cluster of *P. clarkii* aquaculture.

It should be noted that the 60-day duration reflects early plastic responses rather than long-term adaptation. Future studies spanning full grow-out cycles (4–6 months) or multiple generations are needed to evaluate the persistence, reversibility, or heritability of the observed trait clusters.

## 5. Conclusions

This study demonstrates that the distinct conditions of intensive pond monoculture and integrated rice–crayfish co-culture systems are associated with two distinct, coherent phenotypic–physiological syndromes in *Procambarus clarkii*, representing a fundamental life-history trade-off. The pond environment promotes a growth-oriented syndrome, characterized by a compact morphology, lipid-anabolic metabolism, and rapid biomass accumulation, albeit at the cost of higher physiological stress and oxidative damage. Conversely, the rice paddy environment fosters a short-term stress-resistance trait cluster-oriented syndrome, featuring a streamlined body shape, enhanced antioxidant and immune defenses, a metabolic shift towards carbohydrate utilization, and lower systemic stress, indicative of superior adaptation to a complex agro-ecosystem. These organismal-level strategies are underpinned by coordinated transcriptomic reprogramming in the hepatopancreas, including the upregulation of key pathways in lipid metabolism and endocrine regulation (*SULT1E1*).

Our integrated multi-level analysis provides a mechanistic understanding of environmental adaptation in crayfish, highlighting that the integrated conditions of different aquaculture systems are linked to animal health, resilience, and physiological trade-offs. These findings offer valuable insights for optimizing sustainable aquaculture practices, suggesting that integrated systems enhance stock robustness, while intensive systems may require management strategies to mitigate physiological load for long-term sustainability and welfare.

Future directions should include the following: (i) long-term and transgenerational studies to determine whether these syndromes are reversible or heritable; (ii) direct challenge experiments (e.g., with pathogens, thermal stress, or pollutants) to test the functional consequences of each syndrome for survival and disease resistance; (iii) selective breeding programs that use molecular markers such as *SULT1E1* and other stress-responsive genes identified herein to develop genotypes that better balance growth and resilience for different production environments; and (iv) fully crossed experimental designs with feeding-matched controls to disentangle the specific contributions of nutrition and environmental structure to the observed trade-off.

## Figures and Tables

**Table 1:** Comparison of morphological characteristics of crayfish under different culture systems.

Culture Type	Replicate	Body Length (cm)	Total Length (cm)	Body Weight (g)
Rice Paddy Culture	1	8.80 ± 0.57	10.10 ± 0.55	25.30 ± 3.63
	2	8.70 ± 0.44	10.10 ± 0.47	25.70 ± 3.88
	3	8.70 ± 0.52	10.30 ± 0.54	35.10 ± 5.68
Pooled (*n* = 90)		8.73 ± 0.52	10.17 ± 0.54	28.70 ± 6.67
Pond Intensive Culture	4	8.90 ± 0.58	10.50 ± 0.59	38.50 ± 7.02
	5	9.20 ± 0.56	10.70 ± 0.64	38.30 ± 5.77
	6	9.10 ± 0.57	10.60 ± 0.69	37.70 ± 6.76
Pooled (*n* = 90)		9.07 ± 0.59	10.60 ± 0.66	38.17 ± 6.72
*p*-value		<0.001	<0.001	<0.001

Note: Data are presented as mean ± SE (*n* = 90 per group). Differences between the two culture modes were analyzed using an independent samples *t*-test.

**Table 2:** Comparison of cephalothorax morphology and shape indices of crayfish under different culture systems.

Culture Type	Replicate	Carapace Length (cm)	Carapace Width (cm)	Carapace Height (cm)	Width/Length Index	Height/Length Index
Rice Paddy Culture	1	5.00 ± 0.36	2.17 ± 0.23	2.23 ± 0.23	0.435 ± 0.040	0.447 ± 0.037
	2	5.12 ± 0.30	2.31 ± 0.20	2.27 ± 0.21	0.452 ± 0.034	0.444 ± 0.034
	3	4.68 ± 0.98	2.15 ± 0.22	2.38 ± 0.22	0.472 ± 0.097	0.526 ± 0.108
Pooled (*n* = 90)		4.93 ± 0.67	2.21 ± 0.22	2.29 ± 0.23	0.453 ± 0.067	0.472 ± 0.074
Pond Intensive Culture	4	4.52 ± 0.99	2.35 ± 0.26	2.45 ± 0.25	0.539 ± 0.122	0.567 ± 0.124
	5	4.41 ± 0.97	2.26 ± 0.22	2.46 ± 0.23	0.527 ± 0.122	0.581 ± 0.138
	6	4.52 ± 1.01	2.33 ± 0.24	2.49 ± 0.24	0.533 ± 0.122	0.577 ± 0.132
Pooled (*n* = 90)		4.48 ± 0.99	2.31 ± 0.24	2.47 ± 0.24	0.533 ± 0.122	0.575 ± 0.131
*p*-value		0.001	<0.001	<0.001	<0.001	<0.001

Note: Data are presented as mean ± SE (*n* = 90 per group). The Width/Length Index was calculated as (Carapace Width/Carapace Length); the Height/Length Index was calculated as (Carapace Height/Carapace Length). Differences between the two culture modes were analyzed using an independent samples *t*-test.

**Table 3:** Comparison of abdominal segment morphology and shape indices of crayfish under different culture systems.

Culture Type	Replicate	Abdominal Length (cm)	Abdominal Width (cm)	Abdominal Height (cm)	Width/Length Index	Height/Length Index
Rice Paddy Culture	1	3.92 ± 0.67	1.27 ± 0.23	1.85 ± 0.21	0.333 ± 0.072	0.484 ± 0.076
	2	3.43 ± 0.44	1.54 ± 0.23	1.89 ± 0.19	0.453 ± 0.074	0.557 ± 0.071
	3	3.69 ± 0.45	1.86 ± 0.22	1.28 ± 0.19	0.506 ± 0.055	0.348 ± 0.050
Pooled (*n* = 90)		3.68 ± 0.58	1.56 ± 0.31	1.67 ± 0.33	0.431 ± 0.103	0.463 ± 0.108
Pond Intensive Culture	4	3.73 ± 0.51	1.98 ± 0.22	1.22 ± 0.18	0.536 ± 0.073	0.332 ± 0.055
	5	3.81 ± 0.24	1.98 ± 0.18	1.21 ± 0.12	0.520 ± 0.044	0.318 ± 0.031
	6	3.77 ± 0.29	2.07 ± 0.24	1.22 ± 0.12	0.552 ± 0.068	0.325 ± 0.036
Pooled (*n* = 90)		3.77 ± 0.36	2.01 ± 0.22	1.22 ± 0.14	0.536 ± 0.064	0.325 ± 0.042
*p*-value		0.184	<0.001	<0.001	<0.001	<0.001

Note: Data are presented as mean ± SE (*n* = 90 per group). The Width/Length Index was calculated as (Abdominal Width/Abdominal Length); the Height/Length Index was calculated as (Abdominal Height/Abdominal Length). Differences between the two culture modes were analyzed using an independent samples *t*-test.

**Table 4:** Hepatopancreatic enzyme activities of *Procambarus clarkii* under different culture systems.

Enzyme Activity	C Group (Pond)	T Group (Rice Paddy)	*p*-Value
Superoxide dismutase (SOD)	71.7 ± 2.3 U/mg protein	98.4 ± 2.7 U/mg protein	*p* < 0.001
Catalase (CAT)	25.0 ± 1.2 U/mg protein	40.6 ± 1.9 U/mg protein	*p* < 0.001
Glutathione S-transferase (GST)	44.9 ± 2.1 nmol/min/mg protein	68.2 ± 2.6 nmol/min/mg protein	*p* < 0.001
Total lipase	180.3 ± 5.1 U/g protein	124.2 ± 4.8 U/g protein	*p* < 0.001
Amylase	84.8 ± 2.4 U/g protein	119.7 ± 3.5 U/g protein	*p* < 0.001
Acetyl-CoA carboxylase (ACC)	8.6 ± 0.4 nmol/min/mg protein	5.0 ± 0.3 nmol/min/mg protein	*p* < 0.001

Note: Data are presented as mean ± SE (*n* = 10). Differences were analyzed using an independent samples *t*-test.

**Table 5:** Hemolymph physiological and immune parameters of *Procambarus clarkii* under different culture systems.

Parameter	C Group (Pond)	T Group (Rice-Paddy)	*p*-Value
Total protein (g/L)	51.9 ± 1.3	46.3 ± 1.1	*p* < 0.001
Glucose (mmol/L)	3.0 ± 0.2	3.1 ± 0.2	*p* > 0.05 (ns)
Total cholesterol (mmol/L)	3.04 ± 0.07	2.40 ± 0.06	*p* < 0.001
Lysozyme activity (U/mL)	44.9 ± 2.3	67.9 ± 2.4	*p* < 0.001
Phenoloxidase activity (U/mL)	12.2 ± 0.6	18.4 ± 0.9	*p* < 0.001
Cortisol (ng/mL)	16.2 ± 1.2	8.3 ± 0.5	*p* < 0.001
Malondialdehyde (MDA) (nmol/mL)	4.89 ± 0.21	3.12 ± 0.13	*p* < 0.001

Note: Data are presented as mean ± SE (*n* = 10). Differences were analyzed using an independent samples *t*-test; ns, not significant.

## Data Availability

All data generated or analyzed during this study are included in this published article and Supplementary Information Files.

## Supplementary Materials

The following supporting information can be downloaded at https://www.mdpi.com/article/10.3390/ani16081217/s1. Table S1: Statistical table of sequencing data; Table S2: Statistical Table of GO Analysis for Differentially Expressed Genes; Table S3: KEGG Pathway Enrichment Summary Table; Table S4: Nutritional composition of the commercial formulated feed and major natural food components in the rice paddy system.
