# Supplementary material for: Contrasting Aquaculture Systems Shape Distinct Growth and Short-Term Stress-Resistance Trait Clusters in the Red Swamp Crayfish
Source: Animals (Basel). 2026 Apr 16;16(8):1217. doi: 10.3390/ani16081217 (PMC13113003; doi:10.3390/ani16081217)
Supplement: Supplementary file 1 [file animals-16-01217-s001.zip › animals-4234810-supplementary.pdf]

SUPPORTING INFORMATION

Gao Gao[1,2,#], Lingyu Gan[1,2], Jingnan Wei[1,2], Hong Luo[4], Huiying Wang[1,2], JiaLong Chen[1,2], Xiaoyi Su[1], Zhangxiu Li[1], Baoliang Bi[1,3]*, Dan Jia[1,2]

1 College of Animal Science and Technology, Yunnan Agricultural University, Kunming 650500, Yunnan, China.

2 Key Laboratory of Plateau Fishery Resources Protection and Sustainable Utilization, Universities of Yunnan Province, Kunming 650500, China

3 International College, Yunnan Agricultural University, Kunming 650500, Yunnan, China.

4 Xishuangbanna Xinheng Ecological Agriculture Technology Co., Ltd., Xishuangbanna 666100, Yunnan, China.

First author： gaogao@ynau.edu.cn(Gao Gao)

* Corresponding author: 2002037@ynau.edu.cn（Baoliang Bi）

Other authors: 1445940659@qq.com(Lingyu Gan), w114425285@163.com(Jingnan Wei), 13600068687@139.com(Hong Luo), 2023210428@stu.ynau.edu.cn（Huiying Wang）, 2024210436@stu.ynau.edu.cn(JiaLong Chen), 2894885478@qq.com(Xiaoyi Su), 758299915@qq.com(Zhangxiu Li), jiadan@ynau.edu.cn(Dan Jia).

Table S1. Statistical table of sequencing data.

Sample	Raw reads	Raw bases	Clean reads	Clean bases	Q20 (%)	Q30 (%)	GC content (%)
G1-1	42412428	6.36E+09	41352806	6.2E+09	97.67	97.5	43.91
G1-2	41447164	6.22E+09	40465300	6.07E+09	97.77	97.63	44.01
G1-3	44952854	6.74E+09	43818482	6.57E+09	97.69	97.48	44.52
G1-4	42099954	6.31E+09	41200116	6.18E+09	97.69	97.86	42.77
G1-5	44859630	6.73E+09	43828562	6.57E+09	97.68	97.7	44.07
G1-6	44685024	6.7E+09	43558624	6.7E+09	97.6	97.48	43.59
G2-1	46001906	6.9E+09	44796872	6.9E+09	98.76	97.38	47.2
G2-2	43795282	6.57E+09	42669600	6.57E+09	98.58	97.43	46.35
G2-3	42914290	6.44E+09	41916418	6.44E+09	98.81	97.67	46.25
G2-4	47859864	7.18E+09	46585796	7.18E+09	98.41	97.34	44.06
G2-5	45134844	6.77E+09	43976134	6.6E+09	98.76	97.43	46.19
G2-6	44193796	6.63E+09	43037600	6.46E+09	98.79	97.38	45.33
G3-1	44109684	6.62E+09	43037776	6.46E+09	98.62	97.57	42.77
G3-2	41306196	6.2E+09	40238970	6.04E+09	98.8	97.42	44.87
G3-3	47084216	7.06E+09	46018710	6.9E+09	98.79	97.74	45.33
G3-4	45632042	6.84E+09	44473076	6.67E+09	98.75	97.46	45.35
G3-5	47163580	7.07E+09	46035954	6.91E+09	98.2	97.61	45.55
G3-6	42533354	6.38E+09	41455256	6.22E+09	98.76	97.47	45.86
G4-1	43439802	6.52E+09	42339524	6.35E+09	98.74	97.47	46.09
G4-2	41458402	6.22E+09	40509632	6.08E+09	97.59	96.03	45.03

G4-3	40974468	6.15E+09	40146860	6.02E+09	98.8	96.2	46.28
G4-4	39452224	5.92E+09	38501300	5.78E+09	98.75	96.06	46.03
G4-5	41765222	6.26E+09	40801784	6.12E+09	98.72	95.93	46.84
G4-6	42647528	6.4E+09	41616044	6.24E+09	98.76	96.05	46.38
G5-1	42002724	6.3E+09	41057320	6.16E+09	97.58	96	45.32
G5-2	39762934	5.96E+09	38801858	5.82E+09	98.77	96.1	45.59
G5-3	43071066	6.46E+09	42005492	6.3E+09	98.8	96.18	46.11
G5-4	43873088	6.58E+09	42835406	6.43E+09	98.82	96.25	46.08
G5-5	47821942	7.17E+09	46834522	7.03E+09	97.96	96.54	45.36
G5-6	47632548	7.14E+09	46549042	6.98E+09	98.11	95.42	45.29
G6-1	43816830	6.57E+09	42797168	6.42E+09	98.83	96.28	46.61
G6-2	42349748	6.35E+09	41402168	6.21E+09	98.84	96.33	45.68
G6-3	41709832	6.26E+09	40776156	6.12E+09	98.74	95.97	46.52
G6-4	39958066	5.99E+09	38978160	5.85E+09	98.76	96.05	46.56
G6-5	40688186	6.1E+09	39694666	5.95E+09	98.81	96.2	46.24
G6-6	43616164	6.54E+09	42461056	6.37E+09	98.46	96.02	46.48

Table S2. Statistical Table of GO Analysis for Differentially Expressed Genes.

Ontology	Term_name	Up_Count	Up_Percent	Down_Count	Down_Percent
molecular_function	binding	744	44	384	45.66
molecular_function	catalytic activity	542	32.05	204	24.26
molecular_function	transporter activity	150	8.87	52	6.18
molecular_function	transcription regulator activity	75	4.44	83	9.87
molecular_function	molecular function regulator activity	72	4.26	38	4.52
molecular_function	molecular transducer activity	57	3.37	34	4.04
molecular_function	ATP-dependent activity	45	2.66	15	1.78
molecular_function	structural molecule activity	29	1.71	10	1.19
molecular_function	molecular adaptor activity	21	1.24	11	1.31
molecular_function	protein folding chaperone	17	1.01	8	0.95
molecular_function	antioxidant activity	13	0.77	1	0.12
molecular_function	toxin activity	5	0.3	4	0.48
molecular_function	cargo receptor activity	7	0.41	2	0.24
molecular_function	cytoskeletal motor activity	7	0.41	2	0.24
molecular_function	molecular carrier activity	2	0.12	4	0.48
molecular_function	translation regulator activity	6	0.35	2	0.24
molecular_function	molecular sequestering activity	2	0.12	1	0.12
molecular_function	small molecule sensor activity	0	0	2	0.24
molecular_function	general transcription initiation factor activity	1	0.06	1	0.12
molecular_function	protein-containing complex destabilizing activity	1	0.06	0	0
cellular_component	cellular anatomical entity	1041	61.56	514	61.12
cellular_component	organelle	561	33.18	293	34.84
cellular_component	membrane	538	31.82	227	26.99
cellular_component	protein-containing complex	219	12.95	115	13.67
cellular_component	cell junction	90	5.32	45	5.35
cellular_component	extracellular region	73	4.32	48	5.71

cellular_component	synapse	44	2.6	19	2.26
cellular_component	membrane-enclosed lumen	49	2.9	16	1.9
cellular_component	supramolecular complex	41	2.42	10	1.19
cellular_component	nucleoid	2	0.12	1	0.12
cellular_component	other organism part	0	0	1	0.12
biological_process	cellular process	817	48.31	366	43.52
biological_process	biological regulation	570	33.71	308	36.62
biological_process	metabolic process	520	30.75	205	24.38
biological_process	developmental process	265	15.67	130	15.46
biological_process	localization	258	15.26	124	14.74
biological_process	cellular component organization or biogenesis	226	13.36	131	15.58
biological_process	response to stimulus	233	13.78	112	13.32
biological_process	multicellular organismal process	182	10.76	94	11.18
biological_process	reproductive process	90	5.32	51	6.06
biological_process	homeostatic process	71	4.2	28	3.33
biological_process	immune system process	45	2.66	33	3.92
biological_process	locomotion	56	3.31	26	3.09
biological_process	biological process involved in interspecies interaction between organisms	43	2.54	28	3.33
biological_process	behavior	41	2.42	18	2.14
biological_process	growth	24	1.42	16	1.9
biological_process	signaling	30	1.77	12	1.43
biological_process	cell population proliferation	28	1.66	8	0.95
biological_process	rhythmic process	22	1.3	8	0.95
biological_process	detoxification	11	0.65	0	0
biological_process	pigmentation	3	0.18	3	0.36
biological_process	viral process	3	0.18	2	0.24
biological_process	reproduction	2	0.12	1	0.12
biological_process	biological process involved in intraspecies interaction between organisms	3	0.18	0	0
biological_process	cell aggregation	0	0	1	0.12
biological_process	cell killing	1	0.06	0	0

Table S3. KEGG Pathway Enrichment Summary Table.

ID	Description	RichFactor	FoldEnrichment	pvalue	qvalue	Significant
map00140	Steroid hormone biosynthesis	0.47826087	4.48440735	7.60E-06	0.00254325	yes
map04146	Peroxisome	0.25842697	2.42313737	3.82E-05	0.00487613	yes
map01212	Fatty acid metabolism	0.30357143	2.84643389	4.37E-05	0.00487613	yes
map00040	Pentose and glucuronate interconversions	0.4	3.75059524	0.00013082	0.010191	yes
map02010	ABC transporters	0.36666667	3.43804564	0.00015222	0.010191	yes
map00061	Fatty acid biosynthesis	0.44444444	4.16732804	0.000265	0.01478411	yes
map00564	Glycerophospholipid metabolism	0.25	2.34412202	0.00040893	0.01955477	yes
map00592	alpha-Linolenic acid metabolism	0.4375	4.10221354	0.0007333	0.03068259	yes
map01040	Biosynthesis of unsaturated fatty acids	0.38095238	3.57199547	0.00092108	0.03425763	yes

Table S4. Nutritional composition of the commercial formulated feed and major natural food components in the rice paddy system.

Component	Crude protein (%)	Crude lipid (%)	Carbohydrates (%)	Crude fiber (%)	Ash (%)	Moisture (%)	Reference
Commercial feed (Tongwei No. 2)	32.5 ± 0.6	6.8 ± 0.3	33.5 ± 1.0	5.2 ± 0.4	12.0 ± 0.5	10.0 ± 0.2	Manufacturer data + our analysis
Plankton (mixed)	28.1 ± 2.5	5.2 ± 0.6	38.5 ± 3.1	3.5 ± 0.5	15.2 ± 1.2	9.5 ± 0.8	[4] and our analysis
Detritus (rice straw)	6.5 ± 0.8	1.2 ± 0.2	65.4 ± 4.2	28.3 ± 2.1	14.6 ± 1.0	10.2 ± 0.5	[4]
Benthic macroinvertebrates (chironomids)	52.3 ± 3.1	12.5 ± 1.1	10.2 ± 1.5	4.2 ± 0.6	6.8 ± 0.7	14.0 ± 1.2	[23]
Periphyton	22.4 ± 1.8	4.8 ± 0.5	45.6 ± 2.9	8.5 ± 0.9	12.5 ± 1.1	6.2 ± 0.6	[24]
Estimated natural diet (weighted average)	16.3 ± 2.1	3.7 ± 0.8	47.3 ± 3.2	14.5 ± 1.8	18.2 ± 2.0	—	Calculated
